# Laparoscopically detected and nonsurgically managed ileal perforation by an ingested fish bone: a case report

**DOI:** 10.1186/s13256-015-0526-7

**Published:** 2015-02-25

**Authors:** Pramodh Chitral Chandrasinghe, Chandrasiri Karapitiya Pathirana

**Affiliations:** 1 Department of Surgery, University of Kelaniya, North Colombo Teaching Hospital, Ragama, 11010 Sri Lanka; 2 District General Hospital, Homagama, 10200 Sri Lanka

**Keywords:** Ileal perforation, Ingested fish bone, Laparoscopy

## Abstract

**Introduction:**

Ileal perforation due to fish bone is a rare event. The condition is difficult to diagnose due to lack of specific clinical features and low sensitivity of imaging techniques. We report a case of ileal perforation by a fish bone that was detected laparoscopically and managed nonsurgically.

**Case presentation:**

A 45-year-old Sinhalese man presented with acute onset right iliac fossa pain and fever for three days. On examination, he had significant right iliac fossa tenderness and guarding. His white cell count and C-reactive protein level were elevated and an ultrasound scan was indicative of a bowel mass formation. A clinical diagnosis of acute appendicitis was made and laparoscopic appendicectomy was scheduled. At initial survey, a thin spike-like structure was retrieved from the bowel mass, which was revealed to be a fish bone. Our patient was managed with antibiotics only and did not develop any complications.

**Conclusions:**

Ileal perforation due to fish bone is a rare condition that can mimic common conditions like appendicitis. Preoperative diagnosis is rarely made. The slow process of fish bone migration results in concomitant sealing of the perforation, reducing contamination. Use of laparoscopy may be useful in diagnosing this condition and preventing the morbidity of laparotomy in these patients.

## Introduction

Perforation of the gastrointestinal (GI) tract due to an ingested fish bone is a rare event occurring in less than 1 percent of patients [1,2]. Diagnosis of this condition is difficult as patients rarely recall the ingestion and none of the imaging techniques can direct toward a definitive diagnosis [3]. They may present with features of localized abdominal sepsis and are commonly suspected as having acute appendicitis. Use of laparoscopy in the management of acute abdominal conditions, both as a diagnostic and therapeutic tool, has increased over the recent past. Although there are few case reports of laparoscopic detection of this condition, those patients had undergone surgery with ileal resection. We report the case of a patient with ileal perforation due to an ingested fish bone who was diagnosed by laproscopy and managed conservatively.

## Case presentation

**Figure 1:**
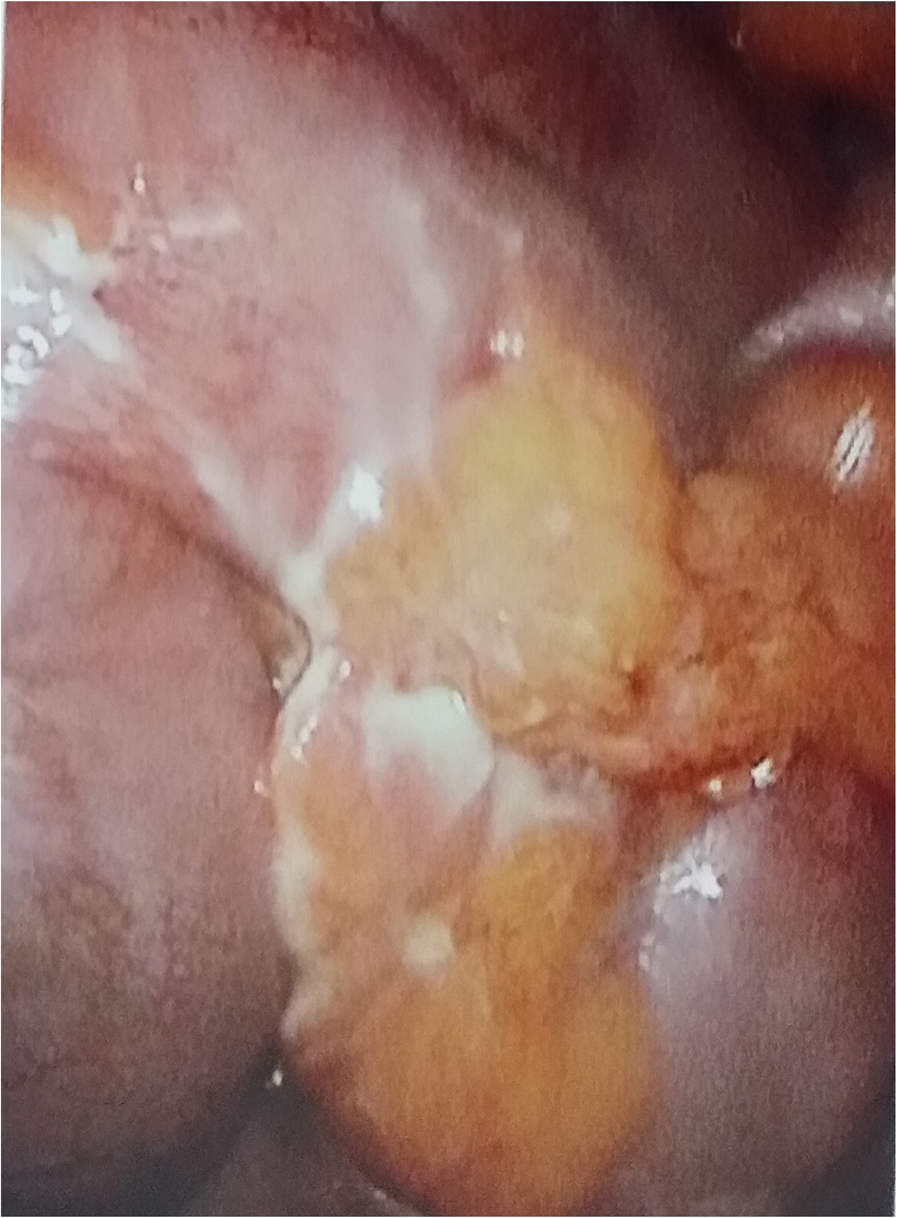
**Laparoscopic image of the bowel and omentum covering the site of the perforation.**

**Figure 2:**
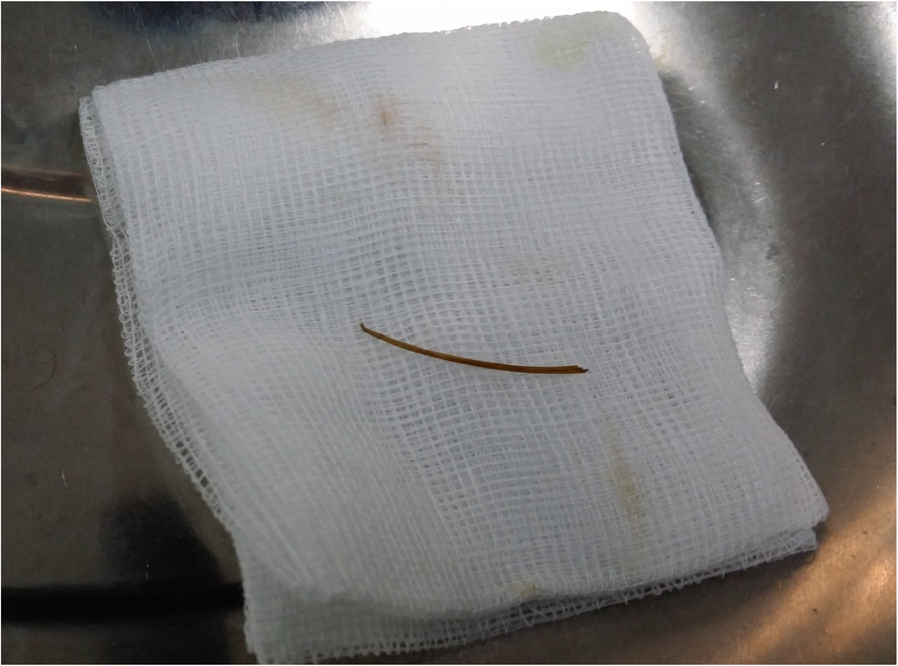
**The retrieved fish bone (kept on a 4cm gauze swab).**

A 45-year-old Sinhalese man presented with a history of right iliac fossa (RIF) pain and fever for three days. He did not have nausea or vomiting and was having normal bowel opening. Our patient had undergone coronary stenting for ischemic heart disease and was on clopidogrel. He was not diabetic. On examination, he was afebrile (37.8ºC) and hemodynamically stable. There was localized tenderness, guarding and rebound tenderness in the right iliac fossa. Clinically, there was no free fluid in the peritoneal cavity. A clinical diagnosis of acute appendicitis was made. His white cell count was 10,800/mm3 with 75% granulocytes and the C-reactive protein level was 45.7mg/L (normal range: 0 to 5mg/L). An ultrasound scan of his abdomen revealed a soft tissue mass formation and localized fluid collection in the RIF suggestive of an appendicular mass. It was decided to proceed with a laparoscopic appendicectomy. Pneumoperitoneum was achieved using the open Hassan technique. A 5mm port was inserted supraumbilically and a 5mm telescope was inserted. On initial exploration of the RIF, a mass formation by ileal loops with purulent exudative membrane around the bowel wall and greater omentum was seen (Figure 1). A thin spike-like structure was protruding from the ileum in close proximity to the mass. After retrieval, it was revealed to be a fish bone that had perforated the terminal ileum (Figure 2). The appendix appeared normal. The mass was not disturbed. It was decided to manage the condition with intravenous cefuroxime 750mg and metranidazole 500mg eight hourly as the perforation was already sealed off. Our patient was free of fever and his bowel movements returned by the second day and he was discharged on oral antibiotics. Our patient was found to be well at a clinic review two weeks after discharge.

## Discussion

The majority of ingested foreign bodies (FBs) pass through the GI tract uneventfully [4]. Fish bones commonly perforate sites with acute angulations such as the ileocecal junction or the flexures of the colon [5]. They may rarely perforate the appendix or a Meckel’s diverticulum [3]. Ileal perforation can result in abscess formation and commonly presents with right iliac fossa pain mimicking acute appendicitis. This patient presented with features of acute appendicitis with mass formation. The clinical, biochemical and ultrasonic findings were favoring the diagnosis of appendicitis. A computed tomography (CT) scan was not performed as it is not a routine investigation in appendicitis. In a majority of previous cases, reported CT scans were performed as a supportive investigation although the sensitivity of CT scans in detecting a fish bone is low [6]. A perforation when detected by CT scan can appear as a segmental intestinal wall thickening, localized pneumoperitoneum, localized fatty infiltration, or associated intestinal obstruction. However, none of these findings is specific, and the definitive diagnosis is made by identification of the calcified FB [6]. The visualization of fish bones depends on the degree of calcification and varies with the species of fish [7]. Perera *et al*. have reported a case of fish bone migration to the liver diagnosed with typical ultrasonic features [8]. This phenomenon occurs when the bone perforates the hepatic flexure. Most of the previously reported cases were managed operatively with resection of small bowel and anastomosis [9,10]. This patient could be managed expectantly as the perforation was already partially sealed off by omentum and fibrinous exudate. An attempt was not made to apply a stitch to the site as the suture would have cut through inflamed tissue and the omental cover would have be disturbed in the process. The peritoneal cavity did not have gross contamination by intestinal content in this patient. This is a well-recognized feature of perforations caused by fish bones as the perforation is caused by impaction and progressive erosion of the FB through the intestinal wall. This also limits the passage of large amounts of intraluminal air into the peritoneal cavity making it difficult to be detected in radiography [5]. The increasing use of laparoscopy for appendicectomy and as a tool for initial exploration of abdominal sepsis has helped in diagnosing this type of rare condition, preventing the morbidity of a laparotomy for patients [11]. This patient was able to be treated nonsurgically as the cause for his symptoms and the extent of sepsis could be accurately ascertained with laparoscopy.

## Conclusions

Fish bone perforation of the ileum is a rare condition that may mimic other common inflammatory conditions. It is difficult to diagnose clinically or with available imaging modalities. The slow process of migration of the bone through the intestine prevents gross contamination of the peritoneal cavity. Increasing use of laparoscopy in managing acute abdominal conditions may help in managing this condition nonsurgically.

## Consent

Written informed consent was obtained from the patient for publication of this case report and any accompanying images. A copy of the written consent is available for review by the Editor-in-Chief of this journal.

## Abbreviations

CT: computed tomography
FB: foreign body
GI: gastrointestinal
RIF: right iliac fossa
